# Interleukin-25-Mediated-IL-17RB Upregulation Promotes Cutaneous Wound Healing in Diabetic Mice by Improving Endothelial Cell Functions

**DOI:** 10.3389/fimmu.2022.809755

**Published:** 2022-01-20

**Authors:** Fang Zhang, Ye Liu, Shiqi Wang, Xin Yan, Yue Lin, Deyan Chen, Qian Tan, Zhiwei Wu

**Affiliations:** ^1^ Department of Burns and Plastic Surgery, Nanjing Drum Tower Hospital, The Affiliated Hospital of Nanjing University Medical School, Nanjing, China; ^2^ Center for Public Health Research, Medical School, Nanjing University, Nanjing, China; ^3^ State Key Laboratory of Analytical Chemistry for Life Science, Nanjing University, Nanjing, China; ^4^ Jiangsu Key Laboratory of Molecular Medicine, Medical School, Nanjing University, Nanjing, China

**Keywords:** IL-25, IL-17RB, diabetes mellitus, wound healing, endothelial cell functions

## Abstract

Diabetic foot ulcer (DFU) frequently leads to non-traumatic amputation and finally even death. However, the mechanism of DFU is not fully understood. Interleukin 25 (IL-25), an alarmin cytokine that responds to tissue injury, has been reported to participate in tissue regeneration and maintaining glucose homeostasis. However, the role of IL-25 in diabetic wound healing remains unknown. Here, we showed that interleukin 17 receptor B (IL-17RB), the functional receptor of IL-25, was significantly inhibited in the wound skin of both diabetic patients with DFU and streptozotocin (STZ)-induced diabetic mice. Topical administration of recombinant IL-25 protein improved angiogenesis and collagen deposition in the wound bed and thus ameliorated delayed diabetic wound healing. IL-25 increased endothelial-specific CD31 expression in diabetic wounds and exogenous IL-25 protected endothelial cells from high glucose-impaired cell migration and tube formation *in vitro*. We further revealed that IL-25-mediated-IL-17RB signaling rescued the downregulation of Wnt/β-catenin pathway both *in vivo* in diabetic mice and *in vitro* in HUVECs and induced the phosphorylation of AKT and ERK 1/2 in HUVECs under high glucose conditions. This study defines a positive regulatory role of IL-25-mediated-IL-17RB signaling in diabetic wound healing and suggests that induction of IL-25-mediated-IL-17RB signaling may be a novel therapeutic strategy for treating poor healing diabetic wounds.

## Introduction

Diabetes mellitus (DM) has been a serious threat to global health regardless of socioeconomic status and national boundaries. It is reported that 463 million people of 20-79 years old suffer from diabetes worldwide in 2019, reaching 9.3% of the age group, and the prevalence is predicted to reach 10.9% (700 million) by 2045 (1, 2). Diabetic foot ulcer (DFU), which affects 19-34% of diabetic patients, is one of the most severe chronic complications of diabetes (3). A study found that patients with DFU had a much higher risk of death than patients without lower extremity wounds, with a 2.5-fold increase and an estimated 5-year mortality rate of 42% for patients with DFU (4). Prolonged non-healing DFU will finally lead to amputation. More than 70% of the patients who undergo diabetes-related amputation will come to the end of their lives within 5 years (3, 5).

Wound healing, a highly organized process consisting of three closely linked phases: inflammation, proliferation and remodeling (6), is dysregulated in patients with diabetes. The occurrence of DFU owes to impaired wound healing caused by multifunctional disorders in all healing phases, among which impaired neovascularization plays a critical role (7, 8). Researches have suggested that diabetes is characterized with endothelial dysfunction and decreased angiogenesis (9, 10). The prolonged hyperglycemic state of diabetic patients leads to disruption of the balance between pro-angiogenic stimulus and anti-angiogenic factors, decrease of endothelial progenitor cells (EPCs) and dysfunction of pro-angiogenic cells such as macrophages (11–13). As a result, insufficient angiogenesis in diabetic wound leads to persistent tissue ischemia and hypoxia, which amplify inflammation and deter wound healing (7).

Interleukin 25 (IL-25), also named interleukin 17E, is a distinct member of IL-17 family which is comprised of six cytokines IL-17A-F (14). Many cells express IL-25, such as activated Th2 cells, eosinophils, mast cells, macrophages, epithelial cells and endothelial cells (15, 16). Unlike other members, IL-25 has only 16-20% homology with IL-17 (17), indicating its unique function. IL-25 signals through a heterodimer receptor formed by IL-17RB and IL-17RA and was reported to participate in tissue regeneration and regulate fibrosis and angiogenesis (15, 18, 19). It was found that IL-25 and its functional receptor IL-17RB were significantly increased in the bronchial mucosa of asthmatic patients, and IL-25 was able to induce airway remodeling and angiogenesis in asthma and played an important role in pulmonary fibrosis (18, 20). Mice with deficiency of IL-25 or IL-17RB had impaired collagen deposition in granulomatous pulmonary inflammation induced by *S. mansoni* egg (21). IL-25 also increased the number, length and area of microvascular structures formed by human umbilical vein endothelial cells (HUVECs) *in vitro* while blockade of IL-17RB rather than IL-17RA inhibited the tube formation induced by IL-25. Furthermore, IL-25 increased IL-17RB mRNA and protein expression and vascular endothelial growth factor (VEGF) mRNA in HUVECs under normal conditions (18, 22). IL-25 also plays a role in metabolic diseases. Nonalcoholic fatty liver diseases (NAFLD) patients with higher body mass index (BMI) had lower IL-25 expression in serum and liver. IL-25 stimulated macrophages to polarize toward the M2 phenotype, promoted lipid metabolism by regulating the expression of lipolytic and lipogenic enzymes and improving mitochondrial respiratory capacity, thereby against obesity (23). Additionally, it was reported that genetic variations of IL-25 and IL-17RB was associated with type 1 diabetes mellitus (T1DM) (24); IL-25 had a function in improving glucose tolerance and stabilizing glucose homeostasis by inducing a series of cells to infiltrate into visceral adipose tissue, including eosinophils, NKT cells, innate lymphoid type 2 cells (ILC2) and alternatively activated macrophages (25). However, whether IL-25 has a role in diabetic wound healing remains unclear.

Here, we explored the roles of IL-25 in diabetic wound healing and investigated possible mechanisms of IL-25 function per se. Specifically, we found significantly decreased IL-17RB mRNA and protein levels in diabetic wound skin, suggesting an inhibition of IL-25-IL-17RB signaling, and exogenous IL-25 could ameliorate impaired diabetic wound healing by improving vascularization and collagen deposition, which may act by activating AKT, ERK 1/2 and Wnt/β-catenin pathways. Thus, we provided evidences suggesting IL-25-mediated-IL-17RB signaling as a new therapeutic target for the treatment of patients who have difficulty in wound healing.

## Materials and Methods

### Human Skin Samples

The human skin wound samples were collected from patients in the Affiliated Drum Tower Hospital of Nanjing University Medical School, including seven normal patients without diabetes and eleven diabetic patients with DFU. The information of all patients is presented in **Supplementary Table 1**. Three to five millimetres of skin was taken from the wound edges of normal and diabetic patients and each skin sample was divided into two parts. One part was fixed and embedded in formaldehyde and then taken for immunohistochemical analysis of IL-17RB or β-catenin, while another part was homogenized and used for RNA isolation. The acquisitions of all human samples and the study protocol were approved by Medical Research Ethics Committee of the Affiliated Drum Tower Hospital of Nanjing University Medical School (#2020-365-02), which were in line with the Declaration of Helsinki Principles. All patients included in the study provided written informed consent.

### Materials and Regents

Recombinant mouse IL-25 (rmIL-25, Cat#: 587306) was brought from Biolegend, recombinant human IL-25 (rhIL-25, Cat#: 8134-IL) was purchased from R&D systems and prepared following instructions. Rabbit anti-CD31 antibody (Cat#: GB11063-2) was obtained from Servicebio. Goat anti-IL-17RB antibody (Cat#: AF1040) was obtained from R&D systems. Rabbit anti-VEGF antibody (Cat#: ab52917) and rabbit anti-β-catenin antibody (Cat#: ab32572) was from Abcam. Mouse anti-GAPDH antibody (Cat#: 60004-1-Ig) and rabbit anti-IL-17RB antibody (Cat#: 20673-1-AP) were obtained from Proteintech. Rabbit anti-phospho-Akt antibody (Cat#: 4060T), rabbit anti-Akt antibody (Cat#: 4091T), rabbit anti-phospho-ERK 1/2 antibody (Cat#: 4370S) and rabbit anti-ERK 1/2 antibody (Cat#: 4695T) were purchased from Cell Signaling Technology (CST). Glucose and streptozotocin (STZ, Cat#: S0130) were purchased from Sigma-Aldrich; and pLenti-CMV-IL-25-GFP-puro lentiviral plasmid (Lenti-IL-25-GFP, Cat#: PPL02165-4a) was obtained from Public Protein/Plasmid Library (PPL). The lentiviral packaging plasmid pMD2.G (Cat#: 12259) and psPAX2 (Cat#: 12260) were obtained from AddGene. Basement membrane matrix (Cat#: 354234) was obtained from Corning.

### Animal Experiments

The animal study was reviewed and approved by the Institutional Animal Care and Use Committee of Experimental Animals in Jiangsu Province and the Ethics Committee of Nanjing University. Six-week-old male BALB/c mice were purchased from Yangzhou University Medical Centre (Yangzhou, China). All the mice were maintained under specific pathogen-free (SPF) environment with free access to food and water. To establish STZ-induced diabetic models, ten mice in each group were intraperitoneally injected with STZ (100 mg/kg) prepared in sodium citrate buffer daily for two consecutive days except for those in the control group. After one week, blood glucose levels were measured and the mice whose blood glucose levels kept steadily over 16.7 mM for two weeks were considered diabetic (26) and used for the subsequent wound experiments. Then, two full-thickness excisional wounds of 6 mm in diameter, with a distance of approximately 10 mm, were performed on the dorsal surface of 1% pentobarbital sodium anaesthetized mice under sterile conditions. On day 0, 1, 2, 4, 6 after injury, mice (n = 10) were injected with sterile phosphate buffered saline (PBS) diluted mouse recombinant IL-25 protein (1 μg for each mouse) that localized around lesion or analogous volume of sterile PBS solution injection as control. The digital images of wound were captured on the day of surgery, the first day and every other day after the first day with a ruler reference beside wound correcting the distance of mice and camera. Wound areas were measured by ImageJ software (National Institutes of Health) and the wound healing rate was identified as the percentage of initial area.

### Histological, Immunofluorescent Staining and Immunohistochemical Analysis

The mice were randomly selected and euthanized on day 7 after wounding, and harvested wound edge tissues were fixed in 4% paraformaldehyde and subsequently embedded with paraffin or flash-frozen in liquid nitrogen for mRNA and protein expression analysis. Thereafter, paraffin-embedded tissue, 5 μm in thick, was performed for Masson trichrome staining to evaluate the collagen deposition.

For immunofluorescent staining analysis, the skin section, which was dewaxed, hydrated and antigen restored, was washed with PBS and blocked in 2% BSA for 1 h. Then, the tissue was incubated with the primary antibody (rabbit anti-β-catenin antibody, 1:250; goat anti-IL-17RB antibody, 1:200) at 4°C overnight and then washed with 0.1% Tween-20 in PBS (PBST) three times for 5 minutes each followed by staining with Alexa 488 or Alexa 594 secondary antibodies (Thermo Fisher Scientific, USA) for 1 h at room temperature in the dark. After washing, the slides were treated with 1 μg/mL DAPI (Life Technologies) in PBS for 5 min at room temperature to make the nuclei visualized. The collected images (at least three views for each sample) were then measured by using ImageJ software to analyze the fluorescence intensity of IL-17RB or β-catenin in different groups relative to that of DAPI as an internal reference as previously reported (27).

For immunohistochemical assay, the sections were dewaxed, hydrated and quenched the endogenous peroxidase activity with 3% H_2_O_2_ for 10 min and then antigen restored; after washing with PBS for three times, the slices were blocked with 2% BSA for 1 h and incubated with primary CD31 antibody (1:100) overnight at 4°C. Subsequently, after incubating with horseradish peroxidase (HRP)–conjugated secondary antibody at room temperature for 1 h, the slices were colored with DAB substrate solution. Images were captured using an Olympus FluoView FV3000 confocal microscope (Tokyo, Japan).

### Cell Culture

Human umbilical vein endothelial cells (HUVECs), purchased from American Type Culture Collection (ATCC) (Manassas, USA), were cultured in Dulbecco’s Modified Eagle’s medium (DMEM; Thermo Fisher Scientific, Waltham, MA) containing 10% fetal bovine serum (FBS, Gibco) at 37°C with 5% CO_2_. The DMEM containing 5.5 mM glucose was considered as normal glucose (CON) conditions, while high glucose (HG) conditions contained 40 mM glucose, the concentration of which was achieved by adding additional glucose. Different concentrations of rhIL-25 (10, 50, 100, 200, 300, 400 ng/mL) was added into the medium in further experiments.

### RNA Extraction and Real-Time PCR

Total RNA was extracted from cells or homogenized skin tissues using TRIzol reagent (Life Technologies, Carlsbad, USA) following manufacturer’s instruction. Then, total mRNA (1μg per reaction) was reverse-transcribed into cDNA using PrimeScript RT Master Mix for RT-PCR (TaKaRa). Quantitative real-time PCR (qPCR) was performed in triplicate on ABI Prism 7500 Sequence Detection System using the SYBR Green PCR Master Mix (Life Technologies). Housekeeping gene GAPDH and β-actin was used for standardization of mRNA, and analysis was carried out using the 2^-ΔΔCt^ method. The sequences of primer pairs were shown in **Table 1**.

**Table 1:** The sequences of primer pairs used in qPCR.

Gene name	Sequences
Human IL-25	Forward (5’-3’): CAGGTGTACAACCACTTGCC
	Reverse (5’-3’): TCCAGAAATGGGCAGAACTT
Human IL-17RB	Forward (5’-3’): AACAGGCGTCCCTTTCCCTCTGGA
	Reverse (5’-3’): TTCTTGATCCTTTCGTGCCTCCAC
Human β-catenin	Forward (5’-3’): AAAGCGGCTGTTAGTCACTGG
	Reverse (5’-3’): CGAGTCATTGCATACTGTCCAT
Human VEGF	Forward (5’-3’): TACCTCCACCATGCCAAGTG
	Reverse (5’-3’): GATGATTCTGCCCTCCTCCTT
Human IL-9	Forward (5’-3’): GACCAGTTGTCTCTGTTTGGGC
	Reverse (5’-3’): TTTCACCCGACTGAAAATCAGTGG
Human IL-17RA	Forward (5’-3’): TGCCCCTGTGGGTGTACTGGT
	Reverse (5’-3’): GCAGGCAGGCCATCGGTGTA
Human cyclin D1	Forward (5’-3’): TCTACACCGACAACTCCATCCG
	Reverse (5’-3’): TCTGGCATTTTGGAGAGGAAGTG
Mouse IL-17RB	Forward (5’-3’): TGTGTTGGACCATCCACTCT
	Reverse (5’-3’): AGTGTTGCTGATCTTGGCTG
Mouse IL-25	Forward (5’-3’): ACAGGGACTTGAATCGGGTC
	Reverse (5’-3’): TGGTAAAGTGGGACGGAGTTG
Human GAPDH	Forward (5’-3’): TGCACCACCAACTGCTTAGC
	Reverse (5’-3’): GGCATGGACTGTGGTCATGAG
Mouse β-actin	Forward (5’-3’): AAGATCAAGATCATTGCTCCTC
	Reverse (5’-3’): GGACTCATCGTACTCCTG

### Western Blotting

To collect proteins, cell samples or homogenized skin tissues were lysed with RIPA lysis buffer (Santa Cruz, USA). After being centrifuged at 12,000×g for 10 min at 4°C, the cleared lysate was collected, and a BCA protein assay kit (Pierce, Rockford, IL, USA) was used to determine the total protein concentrations. The collected protein was subjected to 10% SDS-polyacrylamide gel for protein separation and transferred onto PVDF membranes (Millipore, Billerica, MA, USA). The membranes were blocked, followed by incubation with respective primary antibodies at 4°C overnight. After washing with PBST (0.1% Tween-20), the membranes were incubated with corresponding secondary antibody (IRDye Fluor 680-labeled IgG or IRDye Fluor 800-labeled IgG (LI-COR, Bioscience)) for 1 h at room temperature and visualized using LI-COR Odyssey Infrared Imager (LI-COR, Bioscience).

### Scratch Wound Migration Test and Tube Formation Assay

HUVECs were grown to confluence in 6-well plate and scratch wound migration test was performed by using 200 μL pipette tip to scratch on the surface of cells in the middle of 6-well plate cultured in normal glucose (CON), high glucose (HG) and high glucose with recombinant human IL-25 protein (HG + rhIL-25) medium, respectively. The wound-healing process was photographed at 0, 12, and 24 h after wounding. The migration rate was quantified by calculating the area of wound closed versus that of the primordial wound.

*In vitro* angiogenesis was evaluated by tube formation assay. Briefly, thawed matrigel (Corning, USA) was added into a pre-cooled 24-well plate at amount of 180 μl per well using pre-cooled pipette tips and incubated at 37°C for 30 min. Then, 5 × 10^4^ per well HUVECs were seeded into the matrigel-coated plate and incubated for another 6 h with different culture mediums (CON, HG, and HG + rhIL-25) at 37°C. Tube formation was observed and imaged with Olympus inverted microscope. Total tube length was quantified using ImageJ software.

### Generation of IL-25 Overexpression HUVEC Cell Line

To generate HUVECs that stably over-express IL-25, the lentiviral packaging plasmid pMD2.G and psPAX2 and pLenti-CMV-IL-25-GFP-puro lentiviral plasmid were co-transfected into 6×10^6^ HEK-293T cells (human embryonic kidney cells) (ATCC) to package virus. The supernatant containing lentivirus was collected and concentrated. 1×10^5^ HUVECs were then infected with 100 μL lentivirus for two days. Subsequently, cells were cultured with medium containing 1.5 μg/mL puromycin for three weeks to select single clones which were used for further experiments to evaluate the expression of proteins, including β-catenin, IL-17RB, p-AKT and p-ERK 1/2.

### Statistical Analysis

Results were presented as means ± SEMs with at least three independent experiments using GraphPad Prisms 6.0 software. The differences among treatment groups were then assessed by student’s *t* test to compare the means of two groups or one-way ANOVA when more than two groups. *P* < 0.05 was considered to be statistically significant.

## Results

### IL-25-Mediated-IL-17RB Signaling Was Involved in Diabetic Wound Healing

**Figure 1:**
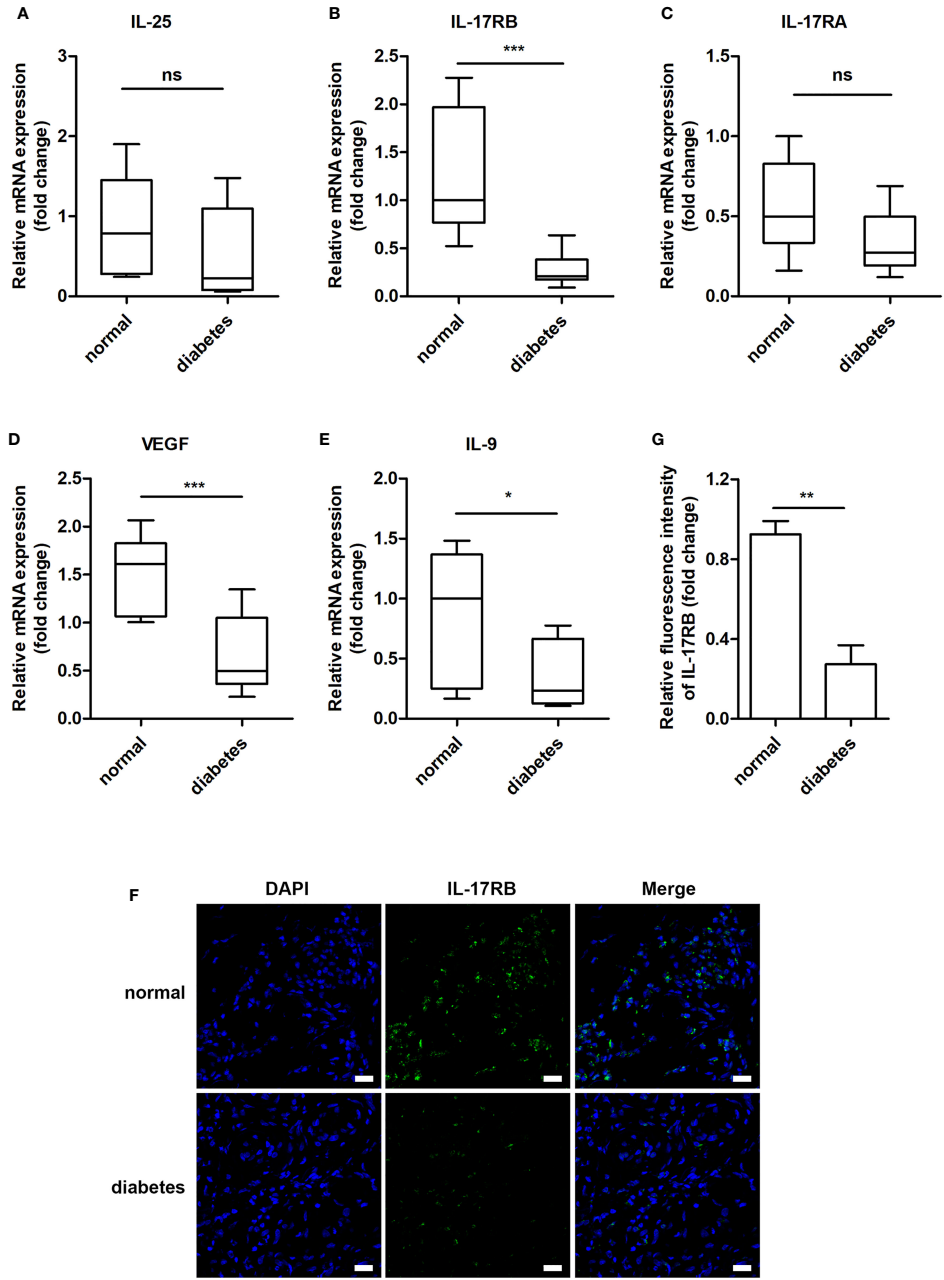
IL-17RB but not IL-17RA is involved in the skin wound healing of patients with DFU. **(A–E)** Relative mRNA expression of IL-25, IL-17RB, IL-17RA, IL-9 and VEGF in the wound edge skin from diabetic patients with DFU (n = 11) and normoglycemic individuals (n = 7), respectively. **(F, G)** Representative immunofluorescent staining for IL-17RB (green) and DAPI (blue) in the wound edge skin from diabetic patients with DFU and normoglycemic individuals. Scale bars, 20 μm. Quantification of the IL-17RB protein in the skin is shown in the histogram in G (n = 3, three views for each sample). *P < 0.05, **P < 0.01, ***P < 0.001. ns, no significance.

Previous studies showed that IL-25 promoted lipid metabolism, thereby fighting against obesity (23), improved glucose tolerance in obese mice and helped to maintain glucose homeostasis (25). Thus, we wanted to know whether IL-25 was involved in the impaired healing of diabetic wound. We firstly analyzed the expression of IL-25, IL-17RA, IL-17RB, and downstream cytokines of the wound edge skin from seven non-diabetic individuals and eleven diabetic patients with DFU and found that IL-25 mRNA was slightly decreased without significant differences between diabetic and non-diabetic wounds (**Figure 1A**); IL-17RB but not IL-17RA mRNA significantly decreased by approximately 78% (*P* < 0.001) in diabetic wounds compared with non-diabetic wounds (**Figures 1B, C**). Consistent with mRNA expression, IL-17RB protein exhibited about 70% reduction (*P* < 0.01) in diabetic wounds as compared with non-diabetic wounds, as shown by immunofluorescent staining assay (**Figures 1F, G**). Moreover, VEGF and interlukin-9 (IL-9) were reported to be the downstream cytokines of IL-25 (18, 22, 28). We found that the mRNA expression of VEGF and IL-9 was also decreased in diabetic wounds compared with non-diabetic wounds, with approximately 56% reduction of VEGF (*P* < 0.001) and 59% reduction of IL-9 (*P* < 0.05, **Figures 1D, E**). The downregulation of VEGF in diabetic wound observed in our study was consistent with previous reports (29, 30).

**Figure 2:**
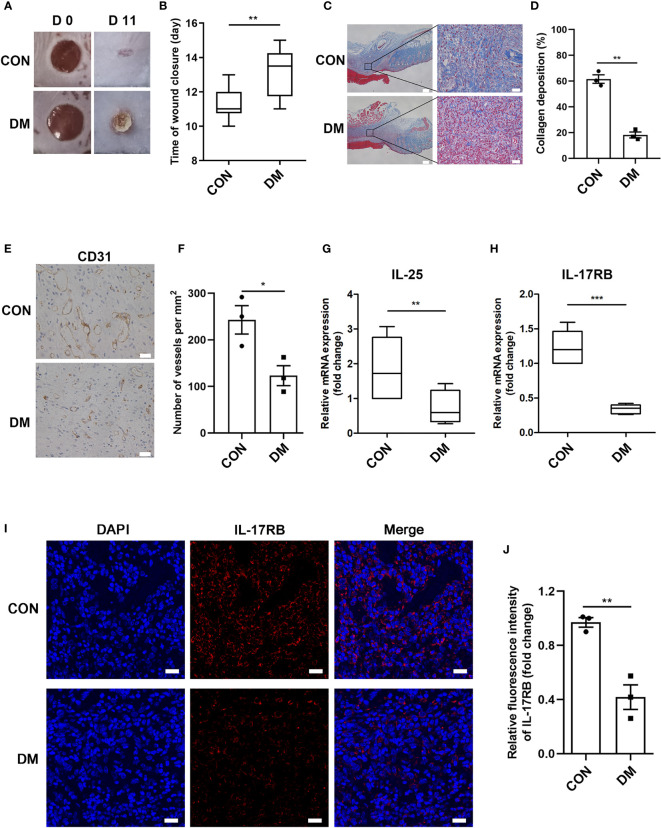
Downregulation of IL-25-mediated-IL-17RB signaling contributes to the delay of wound healing in diabetic mice. 7-day wound biopsies within 2 mm of a wound edge were obtained from dorsal skin of control (CON) and diabetic (DM) mice. **(A)** Images of representative wounds on day 0 and day 11 after injury. **(B)** Quantification of time of wound closure (n = 5). **(C)** Representative images of Masson trichrome staining of dorsal skin sections from control and diabetic mice. Scale bars, 200 μm (left), 25 μm (right). **(D)** Quantification of collagen deposition in different groups (n = 3, three views for each sample). **(E)** Angiogenesis analysis by immunohistochemistry staining of CD31 in dorsal skin sections from control and diabetic mice. Scale bars, 25 μm. **(F)** Quantification of the vessel numbers is displayed in the graph (n = 3). **(G, H)** Relative mRNA expression of IL-25 and IL-17RB in the wound skin from control and diabetic mice (n = 3). **(I, J)** Representative immunofluorescent staining for IL-17RB (red) and DAPI (blue) in skin sections of wound edge from control and diabetic mice. Scale bars, 20 μm. Quantification of the IL-17RB protein expression in the mice skin is shown in the histogram in J (n = 3). *P < 0.05, **P < 0.01, ***P < 0.001.

Using streptozotocin (STZ)-induced diabetic mice, we aimed to investigate whether IL-25 participated in the process of diabetic wound healing. A diabetic mouse model induced by STZ intraperitoneal injection was well established according to the previous studies (26). Then, two full-thickness wounds of 6 mm in diameter were made on the dorsal surface in diabetic and non-diabetic mice. Diabetic mice showed significantly delayed healing compared with non-diabetic mice, with longer time for wound closure (**Figures 2A, B**). Masson trichrome staining of the day 7 wound biopsies also revealed less amount of collagen deposition and lower degree of directional alignment of collagen in granulation tissue in diabetic mice wounds (**Figures 2C, D**), indicating poor extracellular matrix reconstruction and impaired healing of wound. Angiogenic evaluation of the wound skins from diabetic and non-diabetic mice was carried out through immunohistochemistry staining of CD31. The result showed that the wounds of diabetic mice had a poor vascularization with approximately 51% reduction of vessels numbers in the wound bed (*P* < 0.05) (**Figures 2E, F**), indicating an impaired wound healing of diabetic mice. Thereafter, we evaluated the expression of IL-25 and IL-17RB in the wound skin tissues of diabetic and non-diabetic mice. IL-25 and IL-17RB mRNAs were both significantly reduced in diabetic wounds compared with non-diabetic wounds, with approximately 60% reduction of IL-25 mRNA (*P* < 0.01) and 72% reduction of IL-17RB mRNA (*P* < 0.001), respectively (**Figures 2G, H**). Immunofluorescent staining of diabetic wound skins showed 58% reduction (*P* < 0.01) of IL-17RB protein as compared with non-diabetic wound (**Figures 2I, J**), consistent with the results from human wound skin in **Figure 1**. Taken together, we postulated that downregulation of IL-25-mediated-IL-17RB signaling may be associated with delayed healing of diabetic wounds.

### Topical Administration of IL-25 Accelerated Wound Healing in Diabetic Mice

**Figure 3:**
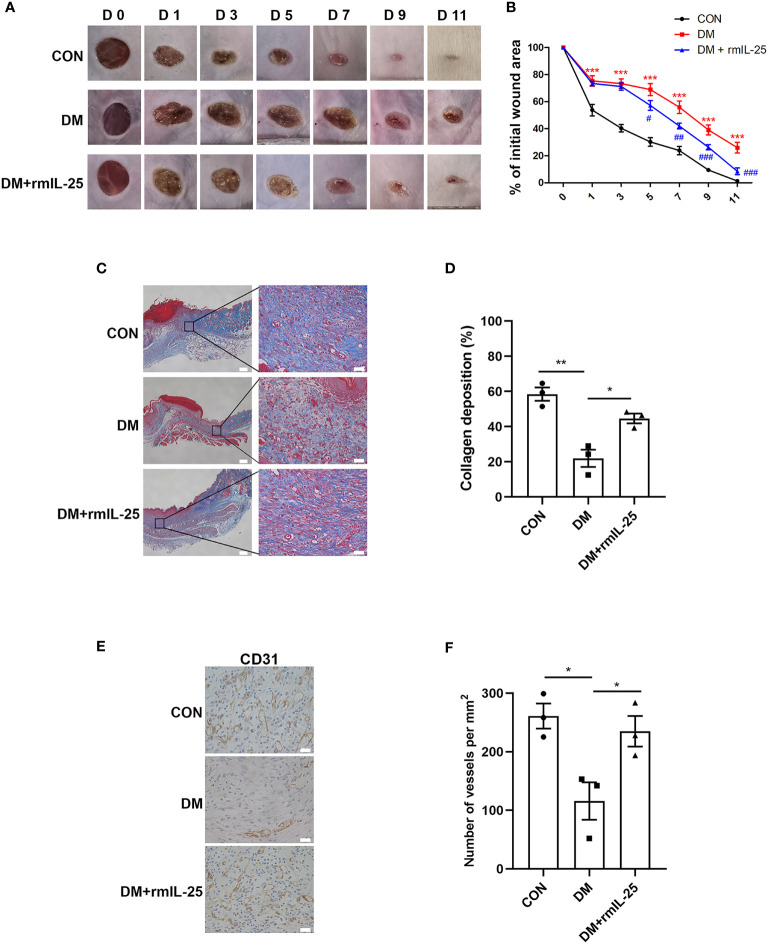
IL-25 promotes wound healing by improving angiogenesis and collagen deposition in diabetic mice. Full-thickness wounds were made on the dorsum of control (CON) and diabetic mice, and the wounds were treated locally with PBS (DM group) or rmIL-25 (DM + rmIL-25 group) on days 0, 1, 2, 4 and 6 after injury. **(A, B)** Wound area analysis showed ameliorated healing in diabetic mice treated with rmIL-25 and the healing is shown as the percentage of the initial wound area. **(A)** Representative wound images at days 0, 1, 3, 5, 7, 9 and 11 during the healing process. **(B)** Quantification results of wound areas are shown (n = 5) (*, DM vs CON group; #, DM + rmIL-25 vs DM group). **(C, D)** Masson trichrome staining of wound skin (day 7 post injury) revealed improved collagen deposition in diabetic mice treated with rmIL-25 **(C)** and the statistical analysis was shown in D (n = 3, three views for each sample). Scale bars, 200 μm (left), 25 μm (right). **(E)** Levels of angiogenesis (vessel density) were evaluated by immunohistochemistry staining for CD31 in wound skin sections (day 7 post injury) from groups of CON, DM and DM + rmIL-25. Scale bars, 25 μm. **(F)** Quantification of the vessel numbers in D is presented (n = 3, three views for each sample). * and ^#^ *P* < 0.05, ** and ^##^ *P* < 0.01, *** and ^###^ *P* < 0.001.

To further evaluate the function of IL-25-IL-17RB signaling during the process of diabetic wound healing, we treated the wounds of diabetic mice with recombinant mouse IL-25 (rmIL-25) dissolved in sterile saline solution with sterile saline solution as a control. Wound area analysis showed a conspicuous amelioration in delayed wound healing in rmIL-25-treated diabetic mice compared with saline-treated diabetic mice, as evidenced by a reduction in wound area from day 5 to day 11 after injury (**Figures 3A, B**). Masson trichrome staining and immunohistochemical staining assays were performed to further analyze the effect of IL-25 on diabetic wound healing. The results showed that topical usage of rmIL-25 improved the collagen deposition, which was significantly reduced in diabetic wound; and the degree of directional alignment of collagens in rmIL-25-treated wounds of diabetic mice was also higher than saline-treated diabetic mice (**Figures 3C, D**), indicating improved tissue remodeling. Immunohistochemical staining of CD31 also revealed a better wound healing in rmIL-25-treated diabetic mice with significant increase (*P* < 0.05) of vessels numbers in the wound bed (**Figures 3E, F**). Thus, we demonstrated that topical treatment of IL-25 significantly accelerated the healing process in diabetic mice.

### IL-25 Reversed the Downregulation of IL-17RB and β-Catenin in Diabetic Wound Tissue

**Figure 4:**
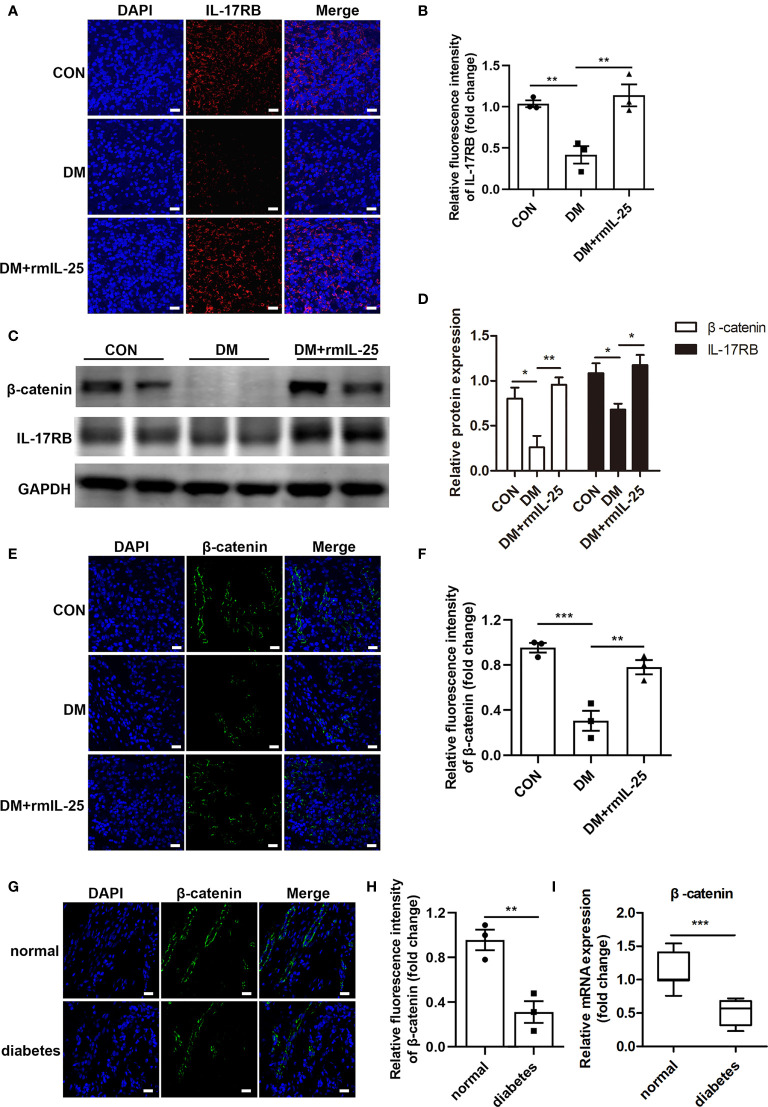
IL-25-upregulated-IL-17RB facilitates wound healing in diabetic mice by rescuing β-catenin expression. **(A)** Representative immunofluorescent staining for IL-17RB (red) and DAPI (blue) in wound edge skin (day 7 post injury) from groups of control (CON), diabetic (DM) and diabetic with rmIL-25 injection mice (DM + rmIL-25). Scale bars, 20 μm. **(B)** Quantification of IL-17RB in A is shown (n = 3). **(C)** Representative western blotting image of β-catenin and IL-17RB in wound skin (day 7 post injury) from groups of CON, DM and DM + rmIL-25. **(D)** Quantification analysis of β-catenin and IL-17RB protein in C is presented (n = 3). **(E, F)** Protein expression level of β-catenin (green) in wound skin (day 7 post injury) from group of CON, DM and DM + rmIL-25 was evaluated by immunofluorescent staining **(E)** and the quantification analysis was shown (n = 3) **(F)**. Scale bars, 20 μm. **(G)** Representative immunofluorescent staining for β-catenin (green) and DAPI (blue) in wound edge skin from diabetic patients with DFU and normoglycemic individuals. Scale bars, 20 μm. **(H)** Quantification analysis of the β-catenin protein expression from G (n = 3). **(I)** β-catenin mRNA expression in the wound edge skin from diabetic patients with DFU (n = 11) and normoglycemic individuals (n = 7) was analyzed by qPCR. *P < 0.05, **P < 0.01, ***P < 0.001.

IL-25 has been reported to increase IL-17RB expression both *in vitro* and *in vivo* (18, 31). Here, we found that IL-17RB protein expression was significantly reduced in the wound bed of diabetic mice and was significantly increased after the topical administration of rmIL-25 (**Figures 4A–D**). Studies have shown that IL-17RB was associated with the up-regulation of β-catenin (32, 33); and Wnt/β-catenin pathway, with β-catenin playing as a key mediator, played an important role in cutaneous tissue repair (34, 35). Thus, the modulation of Wnt/β-catenin pathway was further evaluated. We found abnormal inactivation of Wnt/β-catenin pathway and the expression of β-catenin mRNA decreased by approximately 43% (*P* < 0.001) and β-catenin protein expression decreased by about 67% (*P* < 0.01) in the wound skin of diabetic patients as compared with normoglycemic patients (**Figures 4G–I**). Consistently, in STZ-induced diabetic mice, the protein expression of β-catenin also decreased considerably in comparison with control mice as shown by immunofluorescent staining and western blotting analysis; and the downregulation of β-catenin was significantly reversed in the wound bed in diabetic mice following topical treatment of rmIL-25 (**Figures 4C–F**). Thus, we showed a positive correlation between IL-17RB protein expression and the activation of Wnt/β-catenin signaling in wound tissue.

### IL-25 Promoted VEGF Expression and Improved the Angiogenesis and Migration Capacity of HUVECs Under High Glucose Conditions

**Figure 5:**
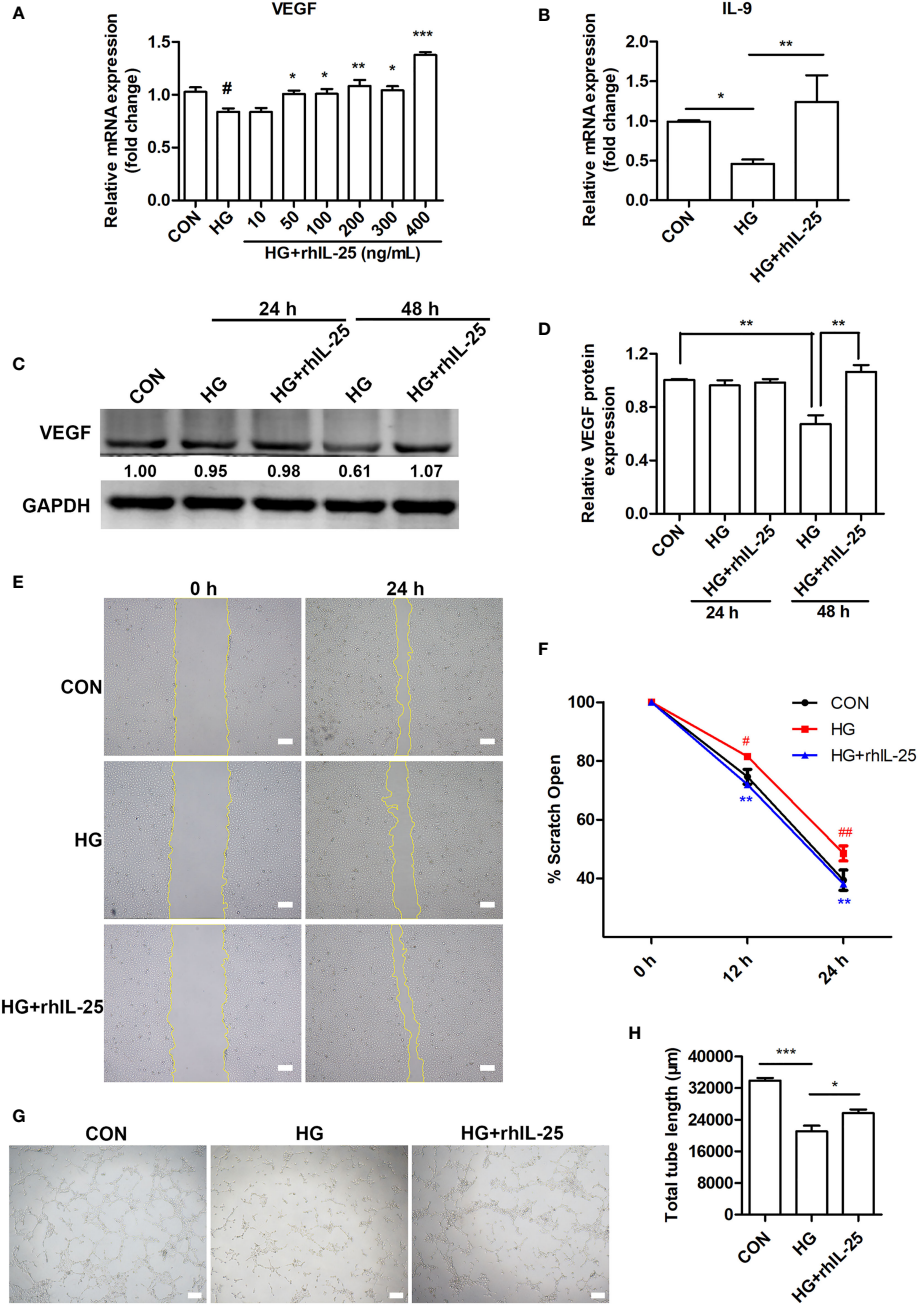
IL-25 promotes angiogenesis and migration of HUVECs under high glucose conditions. **(A)** HUVECs were treated with high glucose and gradients of recombinant human IL-25 (rhIL-25) (10, 50, 100, 200, 300 and 400 ng/mL) for 48 h. Relative VEGF mRNA expression in HUVECs in increasing concentrations of recombinant human IL-25 (rhIL-25) under high glucose conditions was detected by qPCR analysis (n = 3). **(B)** Relative IL-9 mRNA expression in HUVECs stimulated by 400 ng/mL rhIL-25 under hyperglycemic conditions (HG + rhIL-25) (n = 3). **(C)** VEGF protein expression in HUVECs under HG + rhIL-25 treatment was evaluated by western blotting. **(D)** Quantification of VEGF protein expression in C (n = 3). **(E, F)** Representative images and quantification of HUVEC scratch wound migration test (n = 3). Scale bars, 200 μm. **(G, H)** Representative images and quantification of HUVEC tube formation assay (n = 3). Scale bars, 200 μm. * and ^#^ *P* < 0.05, ** and ^##^ *P* < 0.01, ***P < 0.001 (^#^compared with CON group, *compared with HG group). ns, no significance.

Endothelial cell is one of the most important functional cells in skin and plays a critical role in wound healing by participating in angiogenesis (8, 36). We therefore tested the effect of IL-25 on HUVECs under high glucose (HG) conditions by adding recombinant human IL-25 protein (rhIL-25). We observed that HG treatment of HUVECs for 48 h significantly decreased the VEGF mRNA expression, one of the most important pro-angiogenic factors, while rhIL-25 treatment reversed the reduction of VEGF mRNA as compared with those without rhIL-25 treatment (**Figure 5A**). As the highest increase of VEGF was achieved at 400 ng/mL of rhIL-25, this concentration was therefore used for the subsequent experiments. As shown in **Figure 5B**, the mRNA expression of IL-9, a downstream cytokine of IL-25 and having a role in promoting angiogenesis, was significantly increased with the treatment of the cells with 400 ng/mL rhIL-25 under HG conditions for 48 h. Western blotting analysis further revealed that HG also significantly reduced the protein level of VEGF in HUVECs especially when at 48 h, which was reversed by the treatment with rhIL-25 (**Figures 5C, D**). To further investigate the function of IL-25, we performed tube formation assay and scratch wound migration test to evaluate the changes of endothelial function under HG conditions with or without the presence of IL-25. As shown in **Figures 5E, F**, a considerable migration delay was observed under HG conditions and the rhIL-25 treatment expedited the migration. Furthermore, rhIL-25 also increased the total tube length formation, which was markedly reduced under HG conditions (**Figures 5G, H**). Thus, IL-25 improved the function of endothelial cells *in vitro*.

### IL-25 Activated Wnt/β-Catenin Pathway Inhibited Under HG Conditions

**Figure 6:**
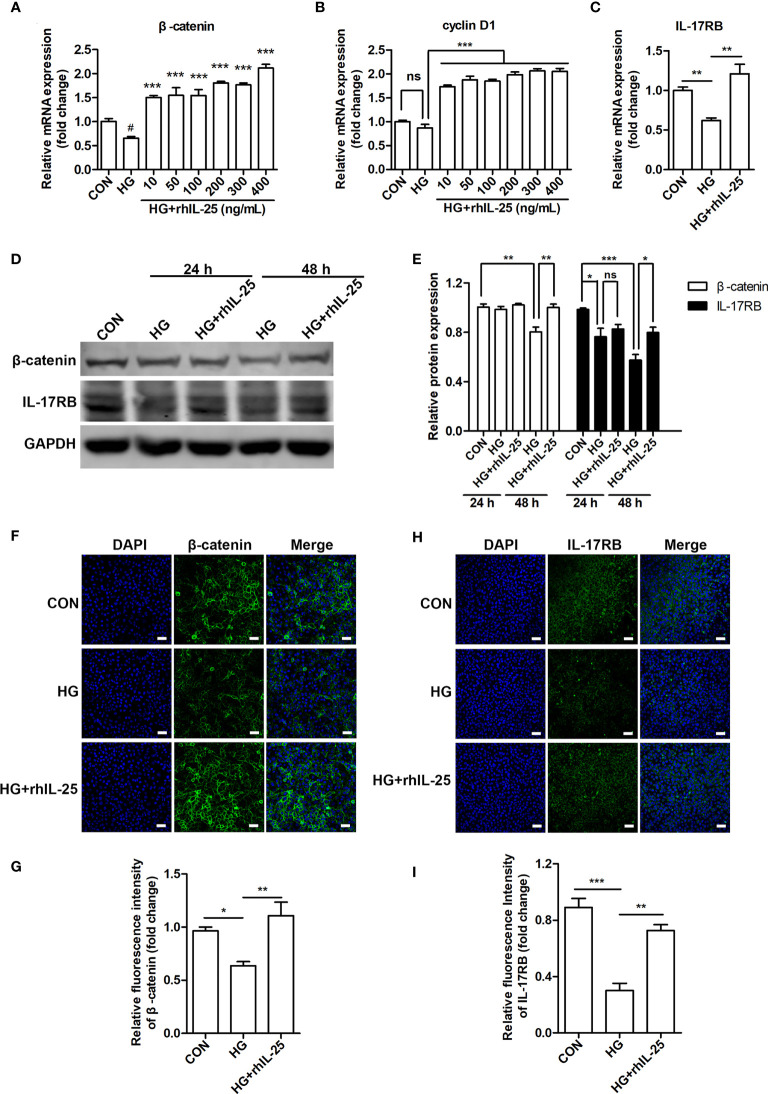
IL-25 rescues the inactivation of Wnt/β-catenin pathway *in vitro*. **(A, B)** HUVECs were stimulated with increasing concentrations of rhIL-25 for 48 h under high glucose conditions (HG). β-catenin and cyclin D1 mRNA from untreated HUVEC and rhIL-25-treated HUVECs under HG conditions were analyzed by qPCR analysis (n = 3). **(C)** IL-17RB mRNA expression in HUVECs stimulated with 400 ng/mL rhIL-25 under HG conditions for 48 h (n = 3). **(D, E)** Western blotting analysis of β-catenin and IL-17RB and their quantification (n = 3). **(F–I)** Representative immunofluorescent staining images and quantification of β-catenin **(F, G)** and IL-17RB **(H, I)** in HUVECs exposed to high glucose and high glucose with rhIL-25 conditions for 48 h (n = 3). Scale bars, 60 μm. * and ^#^ *P* < 0.05, **P < 0.01, ***P < 0.001 (^#^compared with CON group, *compared with HG group). ns, no significance.

To further investigate the mechanism of IL-25 in promoting wound healing and endothelial function, we measured the mRNA of β-catenin and cyclin D1 (a downstream target gene of Wnt/β-catenin pathway) in hyperglycemic HUVECs at different concentrations of rhIL-25 and showed that rhIL-25 increased β-catenin mRNA expression under HG conditions (**Figure 6A**). In addition, cyclin D1 was slightly decreased when exposed to HG, whereas rhIL-25 significantly upregulated the cyclin D1 mRNA under HG conditions (**Figure 6B**). Meanwhile, the IL-17RB mRNA was also upregulated in the presence of rhIL-25 (**Figure 6C**), consistent with our above observations and the published data. Both β-catenin and IL-17RB were significantly decreased when the cells were exposed to HG for 48 h while the addition of rhIL-25 reversed the downregulation of both as measured by western blotting (**Figures 6D, E**). Immunofluorescent staining at 48 h further illustrated the reduction of β-catenin and IL-17RB protein under HG conditions and rhIL-25 treatment reversed the reduction despite the effect of HG (**Figures 6F–I**), consistent with the observation in diabetic wound tissue.

**Figure 7:**
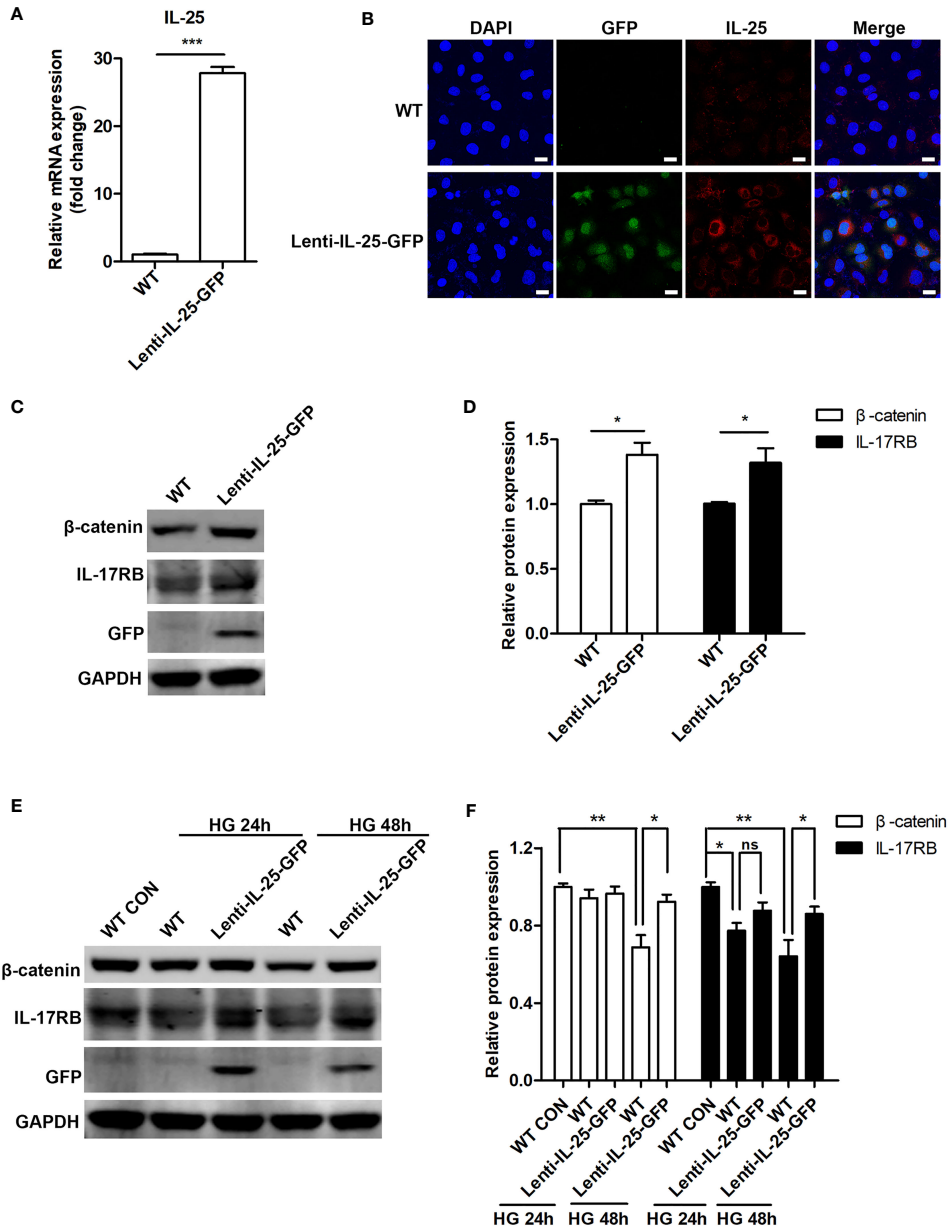
Endogenous overexpression of IL-25 activates Wnt/β-catenin pathway. HUVECs that stably overexpress IL-25 (Lenti-IL-25-GFP-HUVECs) were constructed by using a lentiviral expression vector of human interleukin 25 with the unfused CopGFP protein at the C-terminus (Lenti-IL-25-GFP). The overexpression of IL-25 was verified by q-PCR and immunofluorescent staining assays. **(A)** Relative IL-25 mRNA expression in wild type (WT) and Lenti-IL-25-GFP-HUVECs (n = 3). **(B)** Immunofluorescent staining images of IL-25 (red), GFP (green) and DAPI (blue) in wild type (WT) and Lenti-IL-25-GFP-HUVECs. Scale bars, 20 μm. **(C, D)** Western blotting analysis of β-catenin and IL-17RB in WT and Lenti-IL-25-GFP-HUVECs and their quantification under normal glucose conditions (n = 3). **(E, F)** Western blotting analysis of β-catenin and IL-17RB in WT and Lenti-IL-25-GFP-HUVECs after exposure to high glucose conditions for 24 and 48 h and their quantification (n = 3). *P < 0.05, **P < 0.01, ***P < 0.001. ns, no significance.

We then investigated the effect of IL-25 overexpression on Wnt/β-catenin pathway. As HUVECs were not susceptible to transient transfection of IL-25 plasmids (date not shown), we constructed a HUVEC cell line with stable overexpression of IL-25 by using a lentiviral vector expressing human IL-25 with the unfused CopGFP protein at the C-terminus (Lenti-IL-25-GFP). Firstly, we verified the successful establishment of Lenti-IL-25-GFP-HUVECs by q-PCR and immunofluorescent staining analysis and demonstrated a significant upregulation of IL-25 mRNA and protein (**Figures 7A, B**). Then, we measured the expression of β-catenin and IL-17RB under both normal and high glucose conditions. The results revealed that overexpression of IL-25 was able to not only increase the expression of β-catenin and IL-17RB under normal glucose conditions (**Figures 7C, D**), but also reverse the reduction of β-catenin and IL-17RB when exposed to HG for 48 h comparing with wild type (WT) HUVECs (**Figures 7E, F**).

### IL-25 Induced AKT and ERK 1/2 Phosphorylation in HUVEC Under HG Conditions

**Figure 8:**
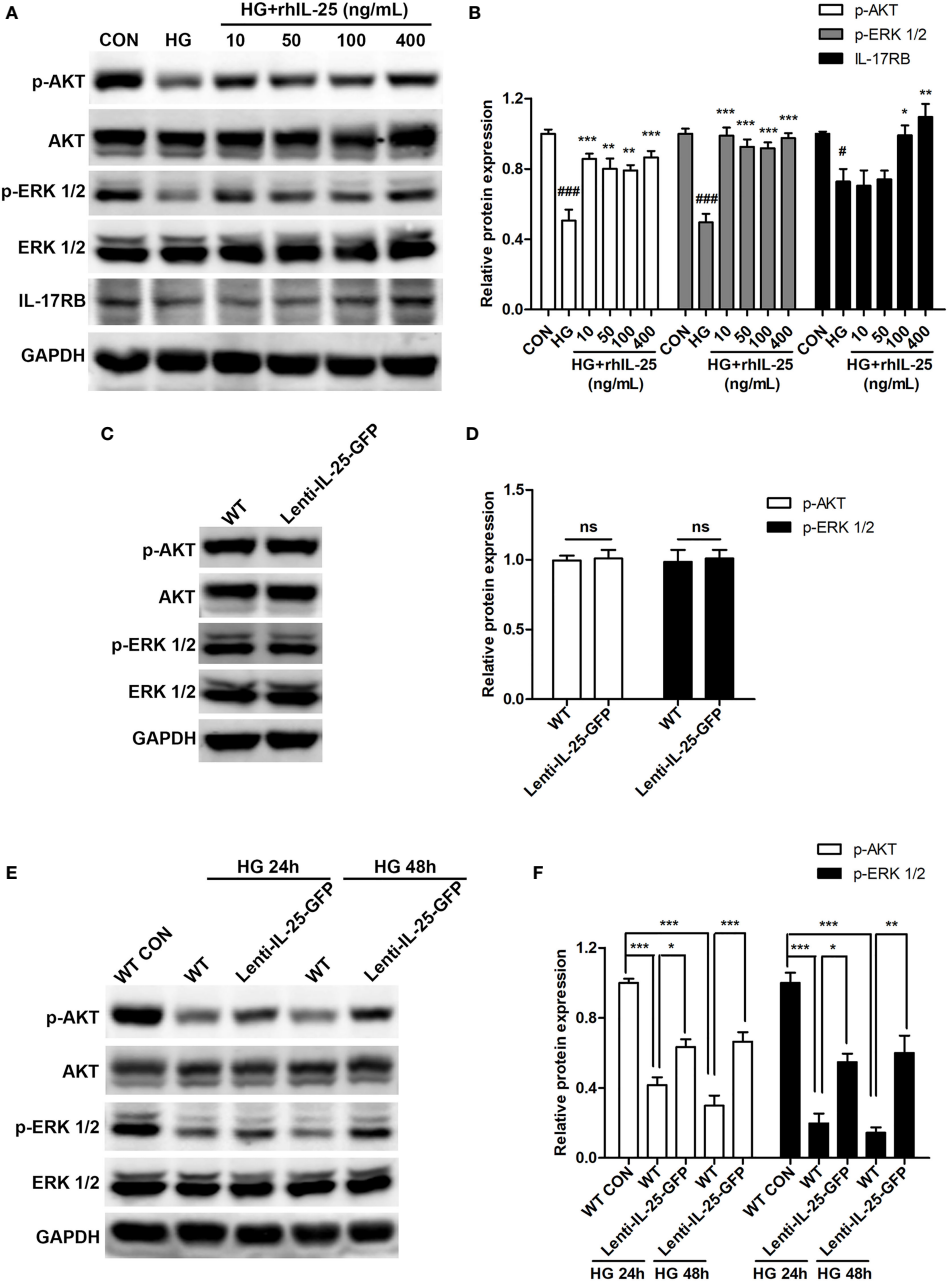
IL-25 induces AKT and ERK 1/2 phosphorylation in HUVECs under high glucose conditions. **(A, B)** HUVECs were cultured in 6-well plate. After grown to 80%, cells were treated with high glucose medium in the presence of a gradient of rhIL-25. Proteins were collected for western blotting analysis after culturing for another 48 h. Representative western blotting images of p-AKT, p-ERK 1/2 and IL-17RB **(A)** and their quantification **(B)** (n = 3) were shown. **(C, D)** WT-HUVECs and Lenti-IL-25-GFP-HUVECs were cultured in normal glucose conditions and proteins were collected for western blotting analysis. Representative western blotting images of p-AKT and p-ERK 1/2 **(C)** and their quantification is displayed in the graph **(D)** (n = 3). **(E, F)** WT-HUVECs and Lenti-IL-25-GFP-HUVECs were treated with high glucose for 24 or 48 h and the proteins were obtained for western blotting analysis. Representative western blotting images **(E)** and quantification **(F)** of p-AKT and p-ERK 1/2 (n = 3) were shown. * and ^#^ *P* < 0.05, **P < 0.01, *** and ^###^ *P* < 0.001 (^#^compared with CON group, *compared with HG group). ns, no significance.

V-akt murine thymoma viral oncogene homolog 1 (AKT) and extracellular signal-regulated kinase 1/2 (ERK 1/2) signaling pathways are known to play important roles in wound healing by regulating cell proliferation, migration, and angiogenesis (37–39) and are reported to be the up-stream signals of β-catenin (33, 40, 41). Our analysis of IL-25 on the activation of the signaling pathways showed that IL-25 was able to induce the phosphorylation of AKT (p-AKT) and ERK 1/2 (p-ERK 1/2) in HUVECs, which were markedly suppressed under HG conditions (**Figures 8A, B**). Parallelly, the activation of these signaling pathways by IL-25 was further determined by endogenous overexpression of IL-25. Under normal conditions, the levels of p-AKT and p-ERK 1/2 were unchanged in Lenti-IL-25-GFP-HUVECs compared with WT-HUVECs (**Figures 8C, D**). However, IL-25 overexpression significantly rescued the down-regulated phosphorylation of AKT and ERK 1/2 when HUVECs were exposed to HG for both 24 h and 48 h (**Figures 8E, F**). These results suggested that IL-25 was capable of reversing the inhibition of AKT and ERK 1/2 signaling pathways induced by HG.

## Discussion

DFU is the most common lower extremity complication of diabetes and frequently leads to amputation (3). Multiple pathophysiological mechanisms result in the occurrence of DFU. Impaired angiogenesis, which leads to insufficient perfusion and hypoxia, plays a vital role in the development of chronic non-healing diabetic ulcers (7, 8). Therefore, HUVECs were chosen in this study to investigate the possible mechanisms involved in the delayed healing of diabetic wound. IL-25 is one of the alarmin cytokines that respond to tissue injury. As numerous evidences point out that IL-25 participates in tissue regeneration and metabolic diseases and IL-25 plays a role in improving glucose tolerance and maintaining glucose homeostasis (15, 18, 19, 23, 25), we attempted to address whether IL-25-mediated signaling participates in the healing process of diabetic wound. In the study, we demonstrated that diabetic mice had delayed recovery, decreased collagen deposition and impaired angiogenesis. Moreover, IL-25-mediated-IL-17RB signaling was down-regulated in diabetic wound skin of both human and mice and exogenous IL-25 improved the healing of diabetic wound by improving collagen deposition and angiogenesis in diabetic mice. *In vitro* analysis revealed that IL-25 was capable of directly increasing the expression of VEGF and IL-9 and stimulating the migration and angiogenesis of HUVECs under HG conditions. Thus, our study brings to light the important role of IL-25-mediated-IL-17RB signaling in impaired diabetic wound healing and the promotional effect of IL-25 on diabetic wound healing, providing a novel therapeutic direction for diabetic patients who have difficulty in wound healing.

Previous studies showed that IL-25 increased vascularity of airways *in vivo* and promoted angiogenesis of human lung vascular endothelial cell and human vascular endothelial cells in normal glucose conditions *in vitro* (18, 22, 42). Here, we found that IL-25 was able to increase the vascularization of diabetic wound and improve the impaired angiogenesis and delayed migration of HUVECs caused by HG. As IL-25 receptor is constitutively expressed in endothelial cells (18, 22), the angiogenic effect can be directly induced by IL-25 administration. IL-25 increased the expression of both VEGF and IL-9 while IL-9 was reported to promote angiogenesis by activating STAT3 pathway (43). Therefore, the angiogenic effect observed in the current study could be the synergistic outcome of both VEGF and IL-9. Furthermore, we observed increased collagen deposition and a more organized directional alignment of collagens in the wound skin of IL-25-treated diabetic mice. IL-25 was reported to increase the expression of a series of pro-fibrotic genes, including collagen I/III, fibronectin, CTGF, α-SMA and TIMP-1; and directly promote human pulmonary fibroblasts proliferate and differentiate to a myofibroblastic phenotype, which may enhance collagen deposition (44, 45). As fibroblast is one of the main functional cells in skin, the increase of collagen deposition induced by IL-25 in diabetic mouse wound skin may be due to the regulation of dermal fibroblast function, which would stimulate more extracellular matrix (ECM) protein deposition. Further investigation is needed to confirm this assumption. In our study, IL-17RB expression was significantly inhibited in diabetic wounds and was up-regulated by topical administration of IL-25. However, the mechanism by which IL-25 and IL-17RB are reduced in diabetic skin wounds and the mechanism of the observed IL-25-induced IL-17RB expression have not yet been revealed at present study and could be an interesting direction to explore for our future research. In addition, it is worth mentioning that IL-25 is known to induce Th2 response, whose activation is accompanied by increased production of typical cytokines such as IL-4, IL-5, IL-9 and IL-13 (46). Th2 response was reported to has a positive role in tissue repair (47) and IL-4 and IL-13 have been reported to activate macrophages, which then activated fibroblasts to regulate the mechanical crosslinking of collagen fibers during skin repair (48). In our study, IL-9, one of the Th2 cytokines was reduced in diabetic wound skin and HUVECs in HG conditions, and was up-regulated after treating HUVECs with rhIL-25. Thus, the pro-healing effect of IL-25 in diabetic wound may also potentially relate to the induction of Th2 response, which deserves further investigation in the future.

In the present study, changes in IL-25 in the wound skin of diabetic patients differed somewhat from those of diabetic mice. IL-25 slightly decreased in diabetic patients, whereas it significantly decreased in diabetic mice. This discrepancy may be due to the impact of clinical treatment, such as debridement treatment of the patients.

The activation of Wnt/β-catenin pathway promotes cutaneous tissue repair by regulating cell proliferation, hair neogenesis, epidermal stem cells (ESCs) function and angiogenesis (34, 49–51). It was reported that wound healing was significantly impaired when Wnt/β-catenin pathway was suppressed, and the healing deficiency was reversed after activating the pathway in diabetic mice and rats (52, 53). Furthermore, it was shown that the mice with enforced expression of β-catenin in invariant natural killer T (iNKT) cell precursors presented particularly exacerbated lung inflammation when treated with IL-25 (54) and IL-17RB was able to induce β-catenin up-regulation thereby promoting lung cancer metastasis (33), indicating a potential regulatory role between IL-25, IL-17RB and β-catenin. Therefore, we speculated that the facilitating effect of IL-25 in diabetic wound healing might be associated with Wnt/β-catenin signal pathway. As expected, we found abnormal suppression of β-catenin in the wound skin of both diabetic patients and mice and the local treatment with IL-25 reversed the inhibition of β-catenin in the wound skin of diabetic mice. Moreover, *in vitro* analysis showed that exposure to HG decreased the expression of β-catenin and its downstream target gene cyclin D1 in HUVECs, which could be rescued by IL-25. Meanwhile, the expression change of IL-17RB exhibited similar trend as β-catenin. Thus, we suggested that IL-25-mediated-IL-17RB signaling promoted diabetic wound healing by inducing the activation of Wnt/β-catenin pathway.

IL-25 is able to activate AKT and ERK1/2 signals in decidual stromal cells (DSCs) (55) and in HUVECs cultured in normal glucose conditions (18). It has been suggested that activation of AKT and ERK 1/2 pathways are able to optimize cellular functions and promote endothelial cell survival and tube formation (38, 56). HG damages endothelial cell function by inhibiting AKT and ERK 1/2 pathways (57, 58). DM down-regulates the activation of AKT and ERK 1/2, resulting in delayed wound healing by decreasing proliferation and increasing cellular apoptosis (59), whereas activation of AKT and ERK 1/2 accelerates not only corneal wound healing but also cutaneous wound closure (37–39). Meanwhile, previous studies suggested that ERK1/2 phosphorylates the Ser9 residue of glycogen synthase kinase-3β (GSK-3β), which results in inactivation of GSK-3β, attenuates GSK-3β-mediated degradation of β-catenin and ultimately leads to β-catenin up-regulation (40). Accordingly, reduction of p-ERK1/2 caused by IL-17RB knockdown leads to reduced expression of β-catenin (33). Moreover, AKT increase the transcriptional activity of β-catenin thus promoting tumor cell invasion and playing a key role in tumor development (41). These studies revealed the role of AKT and ERK 1/2 in the regulation of β-catenin. Thus, we further investigated the effect of IL-25 on these signals in HUVECs under HG conditions. In line with previous reports, we showed that the function of HUVECs was significantly impaired and AKT and ERK 1/2 pathways were inhibited under HG conditions. However, IL-25 significantly ameliorated endothelial function and reversed the down-regulated phosphorylation of AKT and ERK 1/2 in hyperglycemic HUVECs, with similar changes in IL-17RB. Interestingly, although overexpression of IL-25 significantly reversed the reduction of AKT and ERK 1/2 phosphorylation under HG conditions, the phosphorylation of AKT and ERK 1/2 in Lenti-IL-25-GFP-HUVECs did not differ from WT-HUVECs when cultured in normal glucose conditions. We speculate that the discrepancy could be due to the fact that IL-25 is an alarmin cytokine that stimulates the repair process during injury and acts as an autocrine or paracrine (15, 60, 61). Taken together, our study suggested that the activation of Wnt/β-catenin pathway in diabetic wound and hyperglycemic HUVECs may be mediated by AKT and ERK 1/2.

In conclusion, we showed that IL-25-mediated-IL-17RB signaling was inhibited in diabetic wound. IL-25 increased IL-17RB expression and promoted diabetic wound healing by improving angiogenesis and collagen deposition. IL-25 also protected endothelial cells from HG-impaired cell migration and tube formation. Furthermore, IL-25 rescued HG-impaired activation of AKT/ERK/Wnt/β-catenin signaling pathway. These findings suggested that IL-25-mediated-IL-17RB signaling could be considered as a novel target for diabetic wound healing and IL-25 protein could be future therapeutic strategies to protect against delayed diabetic wound healing.

## Data Availability Statement

The original contributions presented in the study are included in the article/**Supplementary Material**. Further inquiries can be directed to the corresponding authors.

## Ethics Statement

The studies involving human participants were reviewed and approved by Medical Research Ethics Committee of the Affiliated Drum Tower Hospital of Nanjing University Medical School. The patients/participants provided their written informed consent to participate in this study. The animal study was reviewed and approved by the Institutional Animal Care and Use Committee of Experimental Animals in Jiangsu Province and the Ethics Committee of Nanjing University.

## Author Contributions

FZ and DC designed the experiments, conducted data analysis and wrote the draft manuscript. FZ and YeL performed the experiments. FZ, SW, XY, and YuL collected clinical samples. DC, QT, and ZW supervised the research project, reviewed research data, interpreted the results, wrote and edited the manuscript. QT and ZW provided funding sources and experimental resources. All authors reviewed the manuscript and approved the final version for publication.

## Funding

This work was supported by National Natural Science Foundation of China (31970149, 81671922 and 81974288).

## Conflict of Interest

The authors declare that the research was conducted in the absence of any commercial or financial relationships that could be construed as a potential conflict of interest.

## Publisher’s Note

All claims expressed in this article are solely those of the authors and do not necessarily represent those of their affiliated organizations, or those of the publisher, the editors and the reviewers. Any product that may be evaluated in this article, or claim that may be made by its manufacturer, is not guaranteed or endorsed by the publisher.
