# Supplementary material for: Interleukin-25-Mediated-IL-17RB Upregulation Promotes Cutaneous Wound Healing in Diabetic Mice by Improving Endothelial Cell Functions
Source: Front Immunol. 2022 Jan 20;13:809755. doi: 10.3389/fimmu.2022.809755 (PMC8810642; doi:10.3389/fimmu.2022.809755)
Supplement: Supplementary file 1 [file Table_1.docx]

Supplementary Material

# Supplementary Tables

Supplementary Table 1 The information of human patients**.**

| Number | Group | Gender | Age | Medication | Diagnosis | Duration of  diabetes (years) | Smoke |
| --- | --- | --- | --- | --- | --- | --- | --- |
| 1 | Diabetes | Female | 73 | Insulin, Amlodipine | T2DM, DFU, Hypertension, Cerebral infarction, Anemia | 5 | - |
| 2 | Diabetes | Female | 66 | Insulin, Metformin, Glipizide | T2DM, DFU, DPN, cerebral infarction, PVD, Pneumonia, Osteomyelitis, Anemia, Chronic cholecystitis and cholelithiasis, Incomplete ileus | 7 | - |
| 3 | Diabetes | Female | 70 | Insulin, Metformin, Irbesartan | T2DM, DFU, Hypertension, Cerebral infarction, Hypoproteinemia | 13 | - |
| 4 | Diabetes | Male | 65 | Insulin, Metformin, Glimepiride, Glipizide, Acarbose, Pioglitazone, Aspirin, Clopidogrel, Atorvastatin calcium, Ticagrelor | T2DM, DFU, Coronary heart disease, Cerebral infarction, | 22 | - |
| 5 | Diabetes | Male | 58 | Insulin, Metformin, Sitagliptin, Acarbose | T2DM, DFU, Hypertension, Lower extremity atherosclerotic occlusive disease | 0.33 (4 months) | Yes |
| 6 | Diabetes | Female | 71 | Metformin, Glimepiride | T2DM, DFU, Cervical vascular stenosis, Fatty liver, Left renal cyst | 8 | - |
| 7 | Diabetes | Male | 54 | Metformin, Glimepiride | T2DM, DFU, Hypertension, Anemia, Hypoproteinemia | 9 | - |
| 8 | Diabetes | Male | 78 | Insulin, Metformin, Compound reserpine tablets, Allisartan | T2DM, DFU, Hypertension, Anemia, Hypoproteinemia, Lower extremity atherosclerotic occlusive disease, Atrial fibrillation, Premature ventricular beats, Chronic renal insufficiency | 10 | - |
| 9 | Diabetes | Female | 43 | Glipizide | T2DM, DFU, Anemia, Hypoproteinemia, Hyponatremia, Hypocalcemia | 12 | - |
| 10 | Diabetes | Female | 63 | Insulin, Metformin, Prednisone | T2DM, DFU, Rheumatoid arthritis, Anemia, Bronchiectasis, Hypoproteinemia, Infectious shock, Soft tissue infection | 7 | - |
| 11 | Diabetes | Female | 66 | Insulin | T2DM, DFU, DPN, Hypertension, cerebral infarction, Osteomyelitis, Anemia, Hyperuricemia, Hypokalemia, Hypoproteinemia, Lower extremity atherosclerotic occlusive disease, Ileus, Type I respiratory failure | 15 | - |
| 1 | Normal | Male | 45 | - | Trauma | - | - |
| 2 | Normal | Male | 29 | - | Trauma | - | - |
| 3 | Normal | Female | 46 | - | Trauma | - | - |
| 4 | Normal | Male | 44 | - | Trauma | - | - |
| 5 | Normal | Male | 48 | - | Trauma | - | - |
| 6 | Normal | Female | 65 | - | Trauma | - | - |
| 7 | Normal | Female | 49 | - | Trauma | - | - |

T2DM: Type 2 diabetes mellitus

DFU: Diabetic foot ulcer

DPN: Diabetic peripheral neuropathy

PVD: Peripheral vascular disease
